# PM_2.5_-Induced Oxidative Stress and Mitochondrial Damage in the Nasal Mucosa of Rats

**DOI:** 10.3390/ijerph14020134

**Published:** 2017-01-29

**Authors:** Zhiqiang Guo, Zhicong Hong, Weiyang Dong, Congrui Deng, Renwu Zhao, Jian Xu, Guoshun Zhuang, Ruxin Zhang

**Affiliations:** 1 Department of Otolaryngology, Huadong Hospital, Fudan University, Shanghai 200040, China; gzqhblove@126.com (Z.G.); hongzhc@163.com (Z.H.); zhaorw007@163.com (R.Z.); 2 Center for Atmospheric Chemistry Study, Department of Environmental Science and Engineering, Fudan University, Shanghai 200433, China; weiyangdongfudan@hotmail.com (W.D.); congruidengfudan@hotmail.com (C.D.); jianxufudan@hotmail.com (J.X.); guoshunzhuangfudan@hotmail.com (G.Z.)

**Keywords:** fine particulate matter (PM_2.5_), nasal mucosa, oxidative stress, mitochondria, inflammatory response

## Abstract

Exposure to PM_2.5_ (particulate matter ≤2.5 μm) increases the risk of nasal lesions, but the underlying mechanisms, especially the mechanisms leading to mitochondrial damage, are still unclear. Thus, we investigated the in vivo effects of PM_2.5_ exposure on the inflammatory response, oxidative stress, the enzyme activities of Na^+^K^+^-ATPase and Ca^2+^-ATPase, and the morphology and function of mitochondria in the nasal mucosa of rats. Exposure to PM_2.5_ occurred through inhalation of a PM_2.5_ solution aerosol. The results show that the PM_2.5_ exposure induced increased levels of malondialdehyde (MDA) and levels of proinflammatory mediators, including interleukin 6 (IL-6), IL-8, and tumor necrosis factor-α (TNF-α). These changes were accompanied by decreases in the activities of total superoxide dismutase (T-SOD), Na^+^K^+^-ATPase, and Ca^2+^-ATPase in rat nasal mucosa. PM_2.5_ significantly affected the expression of specific mitochondrial fission/fusion genes (OPA1, Mfn1, Fis1, and Drp1) in nasal mucosa. These changes were accompanied by abnormal alterations of mitochondrial structures, including mitochondrial swelling, cristae disorder, and even fission resulting from higher doses of PM_2.5_. Our data shows that oxidative damage, inflammatory response, and mitochondrial dysfunction may be the toxic mechanisms that cause nasal lesions after exposure to PM_2.5_.

## 1. Introduction

In recent decades, the development of industry and urbanization in China has resulted in high concentrations of air pollutants in large cities and urban areas [1]. PM_2.5_, which is also known as fine particulate matter, is a group of particulate matter (PM) with an aerodynamic diameter less than 2.5 μm. In particular, the relationship between PM_2.5_ and public health hazards has attracted increasing attention. Toxicological studies are necessary in ambient PM_2.5_ due to properties that are very harmful to humans [2].

The nose is not only a natural pathway for breathing but also designed to act as a sophisticated system for conditioning inspired air, vocal resonance, olfaction, nasal resistance, protection of the lower airway, and ventilation and drainage of the sinuses [3]. The nasal airway remains quite susceptible to infection and inflammation. A damaged nasal mucous membrane will produce discharge, congestion, and swelling. The nasal mucosa may also be a major target for many inhaled toxicants in air pollution. Recently, epidemiological studies have demonstrated that exposure to PM_2.5_ leads to contact with nasal mucosa, which causes nasal inflammation [4] and exacerbates allergic rhinitis [5].

Recent data have indicated that oxidative stress and the inflammatory response may play an important role in the nasal epithelium and contribute to the impairment of nasal epithelial barrier dysfunction following PM_2.5_ exposure in vitro [6]. Mitochondria are unique double-membrane subcellular organelles that provide energy through oxidative phosphorylation and participate in metabolic and genetic functions. Mitochondria are dynamic organelles that balance between fission and fusion. Once the balance is disrupted, mitochondrial dysfunction, which includes a reduced ability to generate adenosine triphosphate (ATP) and higher production of reactive oxygen species (ROS), can make the mitochondrial morphology change. Optic atrophy protein 1 (OPA1) is mainly responsible for mitochondrial inner membrane fusion, while Mitofusin-1 (Mfn1) is thought to mediate the outer membrane fusion. Division of mitochondria is mediated by fission proteins, such as dynamin-related protein 1 (Drp1) and fission mediator protein 1 (Fis1) [7].

Mitochondria are also targeted by environmental pollutants such as PM_2.5_ [8]. Mitochondrial dysfunction may be a critical part of the underlying pathophysiology following exposure to PM_2.5_. Although the nasal airway is the first and primary site of exposure to PM_2.5_, the mechanisms responsible for the effects of PM_2.5_ on nasal injury associated with oxidative stress and mitochondrial damage still need to be fully elucidated. In this study, we investigated the in vivo effects of PM_2.5_ exposure on the inflammatory response, oxidative stress, the enzyme activities of Na^+^K^+^-ATPase and Ca^2+^-ATPase, and the morphology and function of mitochondria in the nasal mucosa of rats.

## 2. Materials and Methods 

### 2.1. PM_2.5_ Sample Preparation and Chemical Analysis

PM_2.5_ was collected as previously described [6]. Briefly, the PM_2.5_ was collected using Whatman 41 filters (Whatman, Maidstone, UK) with TSP/PM_10_/PM_2.5_-2 samplers (Dickel, Beijing, China) at a flow rate of 77.59 L/min. The samplers were on the roof of a five-story building (approximately 20 m above ground) at Fudan University (31.3° N, 121.5° E) in Shanghai, China, from November 2014 to April 2015. Almost no high buildings are around this sampling site. The closest industrial sources are located approximately 10.5 km away and are primarily to the southeast and northwest. This site could be regarded as a representative of the megacity Shanghai, surrounded by the mixing of residential, traffic, construction, and industrial sources [9]. The filters were heated to 200 °C for 24 h before sampling. After sampling, the filters were cut into small pieces and immersed in 0.9% saline followed by sonification for 45 min using a KQ-50B water-bath sonicator (Kunshan Ultrasonic Instruments, Jiangsu, China). The obtained PM_2.5_ suspension was concentrated using a vacuum-freeze dry method, and the final product was weighed and stored at −20 °C. Ten inorganic ions (F^−^, CH_3_COO^−^, HCOO^−^, Cl^−^, NO_3_^−^, SO_4_^2−^, PO_4_^3−^, Na^+^, NH_4_^+^, and K^+^) in PM_2.5_ samples were analyzed with ICS 3000 ion chromatography (Dionex, Sunnyvale, CA, USA). Poly aromatic hydrocarbons (PAHs) in PM_2.5_ were measured with a gas chromatography-mass spectrometer (GC-MS; Agilent, Alpharetta, GA, USA), as previously described [6]. Concentrated PM_2.5_ was diluted with sterile 0.9% saline according to experimental concentrations. The diluted PM_2.5_ solution was stored at 4 °C and used 10 mg/mL of PM_2.5_ for a liquid aerosol generator.

### 2.2. Animal Experiments

Thirty-two female Sprague Dawley rats (4–5 weeks old) that were specific pathogen-free (SPF) were purchased from the Experimental Animal Center of Fudan University (Shanghai, China). The rats were housed in Makcrolon cages with a 12-h light-dark cycle. The experimental protocols were approved by the Institutional Animal Care and Use Committee of Fudan University (certificate number: SYXK-Hu-2014-0029). The rats were randomly divided into four equal groups with eight animals each: (1) a negative control (NC) group; (2) a low concentration of PM_2.5_ exposure (LPM_2.5_) group that was exposed to 200 μg/m^3^ PM_2.5_; (3) a moderate concentration of PM_2.5_ exposure (MPM_2.5_) group that was exposed to 1000 μg/m^3^ PM_2.5_; and (4) a high concentration of PM_2.5_ exposure (HPM_2.5_) group that was exposed to 3000 μg/m^3^ PM_2.5_. The PM_2.5_ inhalation exposure system used in this study was previously described and characterized with modifications [10]. Rats were exposed to PM_2.5_ in a quadrangular chamber (70 × 55 × 45 cm^3^) connected to air pumps (HSENG AS18-2, Beijing, China) with a liquid aerosol generator (HRH-WAG6, Beijing, China), which produced particles with aerodynamic diameters less than 2.5 μm. The particle concentration was measured using a PM_2.5_ detector (PC-3A, Jiangsu, China). A high-efficiency particulate air (HEPA) filter was placed at the outlet of the chamber designated for inside–outside air exchange. The addition of a HEPA filter prevented the PM_2.5_ from exiting the chamber. The NC group was exposed to saline for 3 h/day for 30 consecutive days from Day 0 to Day 29. Three groups of animals were exposed to different concentrations of PM_2.5_ for 3 h/day for 30 consecutive days.

### 2.3. Measurement of T-SOD, MDA, Na**^+^**K**^+^**-ATPase and Ca**^2+^**-ATPase Levels in Rat Nasal Mucosal Tissues

Nasal tissue mitochondrial proteins were extracted with a protein extraction kit (Beyotime, Shanghai, China). The levels of total superoxide dismutase (T-SOD), Na**^+^**K**^+^**-ATPase, and Ca**^2+^**-ATPase as well as the contents of malondialdehyde (MDA) in nasal mucosa supernatants were measured using a kit according to the manufacturer’s instructions (Nanjing Jiancheng Bioengineering Institute, Nanjing, China).

### 2.4. Cytokine Measurement

Blood samples were collected from the abdominal aorta. Serum samples were prepared after incubation in ice-temperature storage and centrifugation at 1800× *g* for 15 min and were stored at −80 °C until analysis. The levels of interleukin 6 (IL-6), IL-8, and tumor necrosis factor-α (TNF-α) in the serum of the tested rats were analyzed with an enzyme-linked immunosorbent assay (ELISA) using a commercial kit according to the manufacturer’s instructions (Gefan Biotech, Shanghai, China).

### 2.5. Real-Time Quantitative RT-PCR

Total RNA was extracted from nasal mucosal tissues using TRIzol reagent (Invitrogen, Shanghai, China) according to the manufacturer’s protocols. The obtained RNA was reverse-transcribed to cDNA using SuperScript III Reverse Transcriptase (Invitrogen). The expression of OPA1, Mfn1, Drp1, and Fis1 were quantified from synthesized cDNA using a SYBR Green assay on a CFX96 Touch™ Real-Time Detection System (Bio-Rad, Hercules, CA, USA). All primers were synthesized by Invitrogen, and their sequences are presented in Table 1. β-actin was used as an endogenous reference. Relative quantitation values (2-^ΔΔCt^) were expressed as fold-change over the controls.

### 2.6. Western Blotting

After the last exposure to PM_2.5_, a protein extraction kit (Beyotime, Shanghai, China) was used to purify mitochondrial proteins. Nasal mucosa total protein for actin from fresh nasal mucosa tissues was determined according to the manufacturer’s protocols. Anti-OPA1, anti-Mfn1, anti-Drp1, and anti-Fis1 primary antibodies were purchased from Santa Cruz (CA, USA). Western blot analyses were performed as described previously [11]. Protein bands were scanned using an ECL detection reagent (GE Healthcare Life Sciences, Piscataway, NJ, USA). Band densities were quantified using Quantity One software.

### 2.7. Histological and Ultrastructural Evaluation

The rats were euthanized using pentobarbital sodium (30 mg/kg·bw, i.p.) 24 h after the last inhalation of PM_2.5_. The nasal septum mucosa were harvested and fixed in 10% formaldehyde then processed routinely, embedded in paraffin, sectioned into 4-µm-thick sections, and stained with hematoxylin and eosin (HE). The histopathological changes were evaluated in tissue sections with an Olympus BX40 microscope. At the same time, another piece of nasal mucosa was minced into small fragments (approximately 1 mm^3^), fixed in 2.5% glutaraldehyde in PBS, and post-fixed in 1% buffered osmium tetroxide. Then, the samples were dehydrated in ethanol, embedded in fresh Epon in capsules, and polymerized at 60 °C for 48 h. Ultrathin sections were double-stained with uranyl acetate and lead citrate, and the sections were then examined and photographed under an accelerating voltage of 0.5–30 kV and a transmission electron microscope (TEM, FEI Tecnai G2 Spirit).

### 2.8. Statistical Analysis

The results were expressed as the mean ± standard deviation (SD) and analyzed with SPSS software (SPSS for Windows 18.0, SPSS, Inc., Chicago, IL, USA). A one-way analysis of variance (ANOVA) with the post hoc test was used to test for significant differences between the groups. *p* < 0.05 was considered to be statistically significant.

## 3. Results

### 3.1. Analysis of PM_2.5_ Chemical Characteristics

The chemical characteristics of the PM_2.5_ used in this study were previously described [6]. The mean levels of NO^3−^, SO_4_^2−^, and NH^4+^ ions in the PM_2.5_ samples reached 386.32, 209.34, and 199.14 μg/mg, respectively. Overall, benzo[b]fluoranthene, phenanthrene, fluoranthene, pyrene, and chrysene represent the most abundant PAH in the PM_2.5_ collected from Shanghai.

### 3.2. Effects of PM_2.5_ on T-SOD, MDA, Na**^+^**K**^+^**-ATPase, and Ca**^2+^**-ATPase Levels in the Nasal Mucosa of Rats

**Figure 1:**
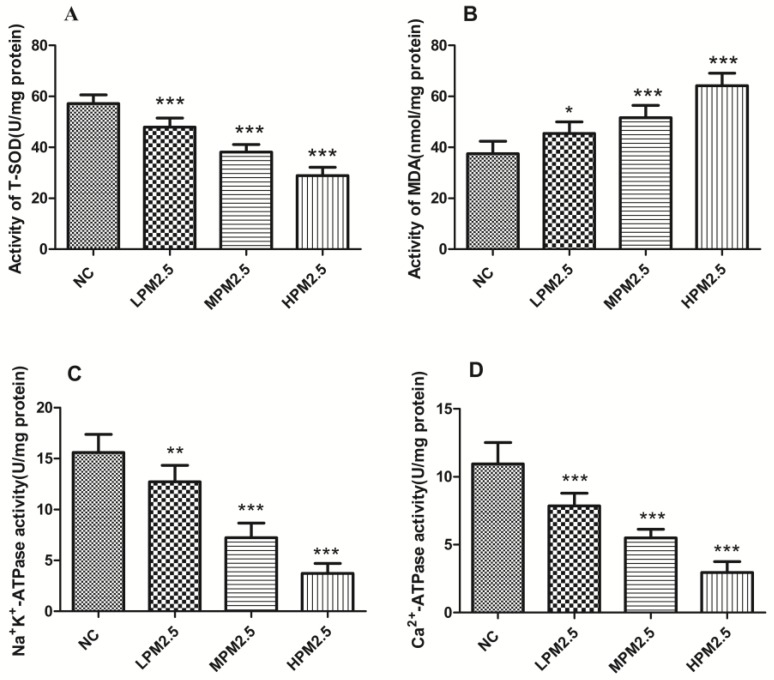
Activity levels for T-SOD (**A**); MDA (**B**); Na^+^K^+^-ATPase (**C**); and Ca^2+^-ATPase (**D**) in the nasal mucosa of rats treated with different PM_2.5_ concentrations. Values are presented as the means ± SD (*n* = 8). * *p* < 0.05, ** *p* < 0.01, *** *p* < 0.001 (LPM_2.5_, MPM_2.5_, HPM_2.5_ group vs. NC group). NC = a negative control group, LPM_2.5_ = exposure to 200 μg/m^3^ PM_2.5_ group, MPM_2.5_ = exposure to 1000 μg/m^3^ PM_2.5_ group, HPM_2.5_ = exposure to 3000 μg/m^3^ PM_2.5_ group.

As indicated in Figure 1, PM_2.5_ at concentrations of 200, 1000, and 3000 μg/m^3^ significantly reduced T-SOD activities and elevated the MDA content in nasal mucosa compared to the control group (*p* < 0.05 or *p* < 0.001). Moreover, PM_2.5_ exposure elicited a notable decrease in Na^+^K^+^-ATPase and Ca^2+^-ATPase activity relative to the control (*p* < 0.01 or *p* < 0.001).

### 3.3. Effects of PM_2.5_ on the Levels of Inflammatory Cytokines

**Figure 2:**
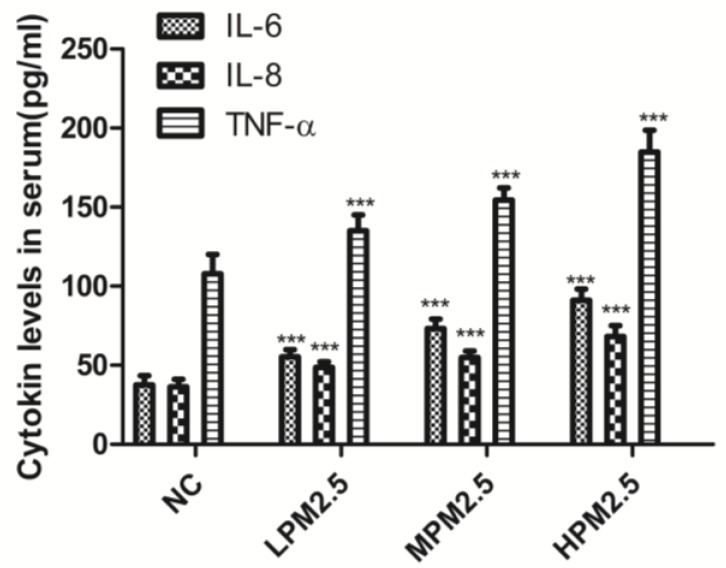
The effects of PM_2.5_ exposure on IL-6, IL-8, and TNF-α in serum. Values are presented as the means ± SD (*n* = 8). *** *p* < 0.001 (LPM_2.5_, MPM_2.5_, HPM_2.5_ group vs. the NC group). NC = a negative control group, LPM_2.5_ = exposure to 200 μg/m^3^ PM_2.5_ group, MPM_2.5_ = exposure to 1000 μg/m^3^ PM_2.5_ group, HPM_2.5_ = exposure to 3000 μg/m^3^ PM_2.5_ group.

Figure 2 shows that the levels of three proinflammatory cytokines (IL-6, IL-8, and TNF-α) had an obvious increase in response to exposure to PM_2.5_ at concentrations of 200, 1000, and 3000 μg/m^3^ compared to the control (*p* < 0.001).

### 3.4. Effects of PM_2.5_ on Rat Nasal Mitochondrial Fusion/Fission Gene Expression

**Figure 3:**
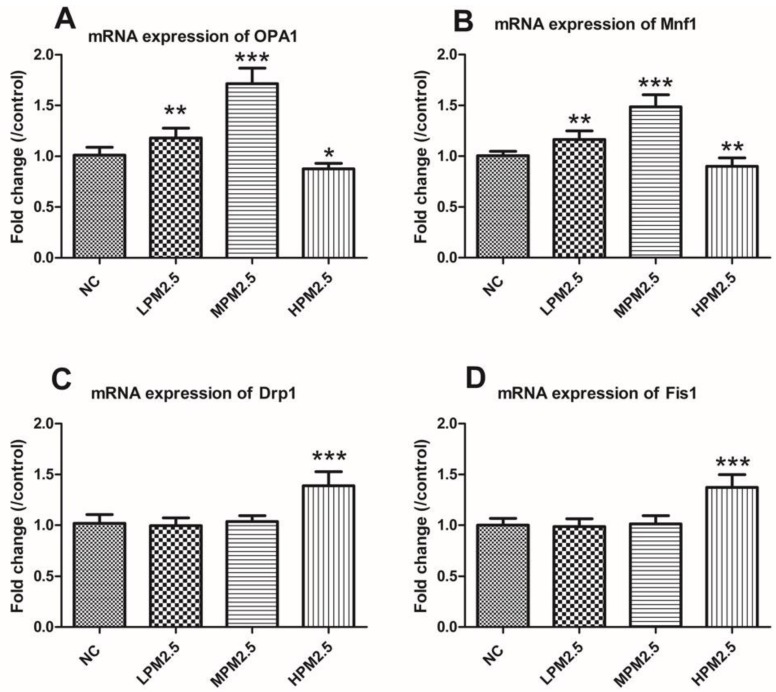
The effects of PM_2.5_ on the mRNA expression of OPA1, Mfn1, Drp1, and Fis1 in rat nasal mucosa according to real-time PCR. Values are presented as the means ± SD (*n* = 8). * *p* < 0.05, ** *p* < 0.01, *** *p* < 0.001 (LPM_2.5_, MPM_2.5_, HPM_2.5_ group vs. NC group). NC = a negative control group, LPM_2.5_ = exposure to 200 μg/m^3^ PM_2.5_ group, MPM_2.5_ = exposure to 1000 μg/m^3^ PM_2.5_ group, HPM_2.5_ = exposure to 3000 μg/m^3^ PM_2.5_ group.

**Figure 4:**
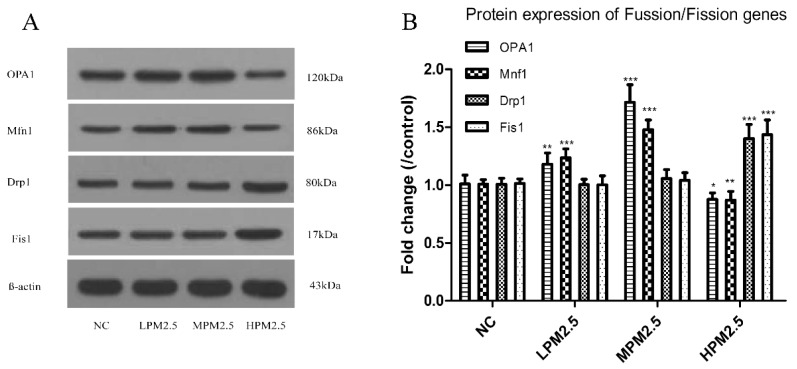
Quantitative analysis of the effects of PM_2.5_ on the expression of OPA1, Mfn1, Drp1, and Fis1 proteins in the nasal mucosa mitochondria of rats. The values are presented as the means ± SD (*n* = 8). * *p* < 0.05, ** *p* < 0.01, *** *p* < 0.001 (LPM_2.5_, MPM_2.5_, HPM_2.5_ group vs. the NC group). NC = a negative control group, LPM_2.5_ = exposure to 200 μg/m^3^ PM_2.5_ group, MPM_2.5_ = exposure to 1000 μg/m^3^ PM_2.5_ group, HPM_2.5_ = exposure to 3000 μg/m^3^ PM_2.5_ group.

As shown in Figure 3 and Figure 4, OPA1 and Mfn1 mRNA as well as protein levels in PM_2.5_-exposed rats significantly increased at concentrations of 200 and 1000 μg/m^3^ PM_2.5_, but the 3000 μg/m^3^ of PM_2.5_ significantly decreased the levels of OPA1 and Mfn1 compared to the control. PM_2.5_ exposure prompted a notable increase in Drp1 and Fis1 mRNA as well as protein levels in the high concentration of PM_2.5_ (3000 μg/m^3^) (*p* < 0.001). However, no difference in Drp1 and Fis1 mRNA as well as protein levels were observed between the low or moderate concentrations of PM_2.5_ (200 and 1000 μg/m^3^) and the control.

### 3.5. Effects of PM_2.5_ on Nasal Mucosa Histology and Ultrastructural Damage

**Figure 5:**
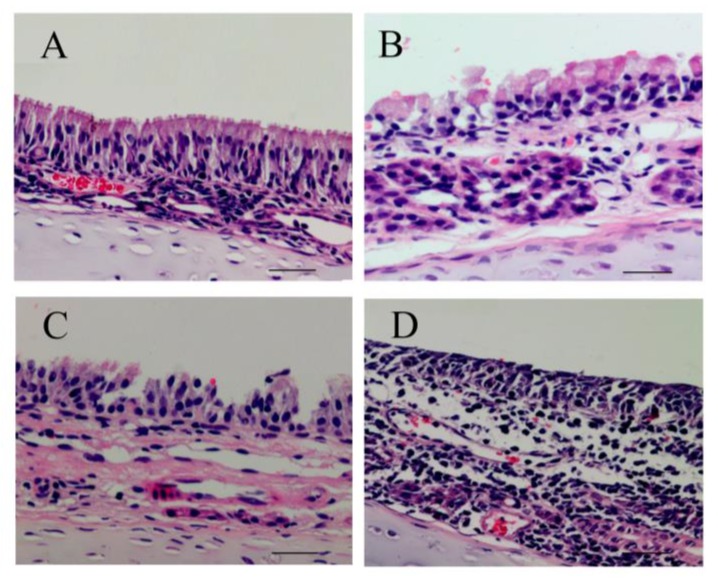
The effects of PM_2.5_ exposure on nasal mucosa histology damage demonstrated with HE staining. (**A**) NC group; (**B**) LPM_2.5_ group; (**C**) MPM_2.5_ group; and (**D**) HPM_2.5_ group (original magnification: (**A**–**D**), ×400, Scale bars = 50 μm). The nasal mucosa from the NC group showed normal morphology, but the nasal mucosa from PM_2.5_-exposed groups showed different degrees of pathological changes. NC = a negative control group, LPM_2.5_ = exposure to 200 μg/m^3^ PM_2.5_ group, MPM_2.5_ = exposure to 1000 μg/m^3^ PM_2.5_ group, HPM_2.5_ = exposure to 3000 μg/m^3^ PM_2.5_ group.

**Figure 6:**
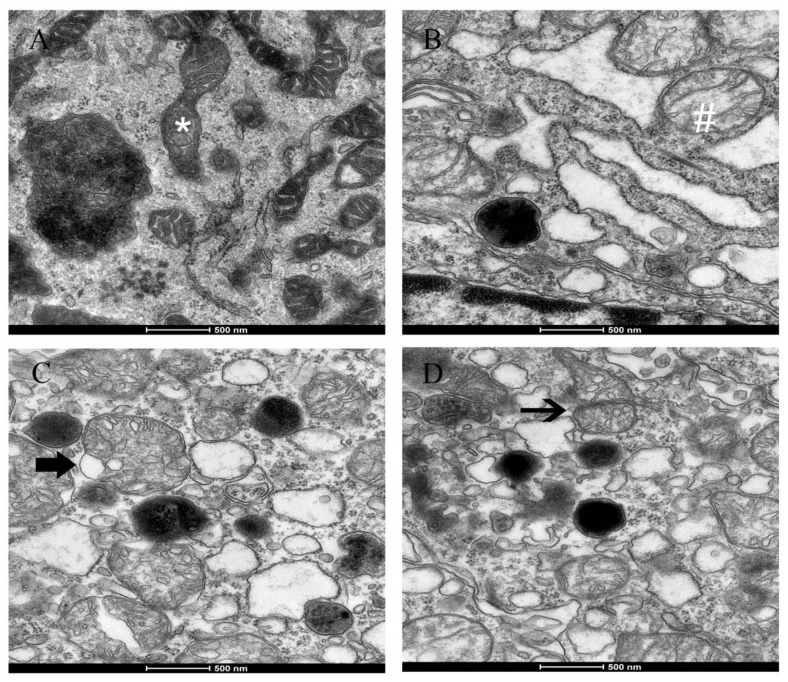
Ultrastructural detrimental effects of PM_2.5_ exposure on nasal mucosa and mitochondria of rats from (**A**) NC group; (**B**) LPM_2.5_ group; (**C**) MPM_2.5_ group; and (**D**) HPM_2.5_ group, 20,000× magnification. Scale bars indicate 500 nm. Normal mitochondria (*) and mitochondrial swelling (#) are shown in (**A**,**B**). The thick arrows indicate sites of mitochondrial crista disorder or vacuolization in (**C**), the thin arrow indicates site of mitochondrial fission in (**D**), respectively. NC = a negative control group, LPM_2.5_ = exposure to 200 μg/m^3^ PM_2.5_ group, MPM_2.5_ = exposure to 1000 μg/m^3^ PM_2.5_ group, HPM_2.5_ = exposure to 3000 μg/m^3^ PM_2.5_ group.

The nasal mucosa from the control group showed no histopathological abnormalities in HE staining (Figure 5A). However, the nasal mucosa of exposure to different concentrations of PM_2.5_ showed morphological alterations. The nasal mucosa of 200 μg/m^3^ PM_2.5_-exposed rats showed a disarray of cilia and vascular congestion (Figure 5B), whereas the nasal mucosa of 1000 μg/m^3^ PM_2.5_-exposed rats showed the nasal epithelium necrosis, a disarray of cilia, vascular congestion, and edema (Figure 5C). Further, the nasal mucosa of 3000 μg/m^3^ PM_2.5_-exposed rats, an absence of cilia, inflammatory cell infiltration (mainly neutrophils and lymphocytes), and submucosal gland hypertrophy were observed (Figure 5D). As the PM_2.5_ concentration increased, the nasal mucosa pathological injuries markedly increased. Figure 6 shows that there were ultrastructural changes in mitochondria from the rat nasal epithelial cells of different groups. Rats in the control group had mitochondrial normal architecture (Figure 6A), whereas mitochondrial swelling and cristae disorder were found in rats exposed to LPM_2.5_ (200 μg/m^3^ PM_2.5_) (Figure 6B). Mitochondrial swelling, membrane breach, and vacuolization were found in rats exposed to MPM_2.5_ (1000 μg/m^3^ PM_2.5_) (Figure 6C). Elevated concentrations of PM_2.5_ (Figure 6D) also led to a more prominent mitochondrial cristae disorder appeared and mitochondrial fission after exposure to a high concentration of PM_2.5_ (i.e., the HPM_2.5_ group).

## 4. Discussion

PM_2.5_ has been linked to a range of adverse health effects, including ear, nose, and throat as well as gastrointestinal, nutritional, renal, and cardiovascular diseases [12]. A better understanding of the mechanisms of nasal injury caused by ambient PM_2.5_ will be helpful for clarifying the pathogenesis of nasal disease as a result of exposure to air pollution. In the current study, we demonstrate that inhalation exposure to PM_2.5_, especially at high concentrations, was associated with significantly increased histology and ultrastructural damage of mitochondria as well as an increase in the MDA content and proinflammatory cytokines levels in serum. Additionally, oxidative stress and ion homeostasis in rat nasal mucosa were also aggravated.

Assuming a minute volume of 200 mL for a 300 g rat [13], inhalation exposure to 200, 1000, and 3000 μg/m^3^ of PM_2.5_ for 3 h/day over 30 consecutive days delivered total amounts of 0.22 mg, 1.08 mg, and 3.24 mg of PM_2.5_, respectively, in this study. The total PM_2.5_ exposure levels in this study therefore corresponded with previous intratracheal instillation studies, which defined a low dose as 0.2 mg/rat, a medium dose as 0.8 mg/rat, and a high dose as 3.2 mg/rat [14]. The levels of PM_2.5_ exposure in our study were 8-(LPM_2.5_), 40-(MPM_2.5_), and 120-(HPM_2.5_) fold higher than air quality guidance by the World Health Organization (WHO) (PM_2.5_, 25 μg/m^3^ over a 24 h period). In the sampling period in Shanghai, China, the mean mass concentration of PM_2.5_ was 52.2 μg/m^3^ in 2014 and was 67.9 μg/m^3^ in 2015, which was dramatically higher than the WHO standard (an annual average of 10 μg/m^3^) [15].

From HE staining results in Figure 5, we observed ciliary loss, vascular congestion, edema, inflammatory cell infiltration (mainly neutrophils and lymphocytes), and submucosal gland hypertrophy in the nasal mucosa of rats exposed to PM_2.5_. To explore whether the nasal mucosa injury induced by PM_2.5_ was related to increased inflammatory activity, we detected the levels of proinflammatory cytokines, such as IL-6, IL-8, and TNF-α, which are known to be higher in the inflammatory response in nasal epithelial cells exposed to PM_2.5_ in vitro [6]. Moreover, PM_2.5_ can increase ROS levels and reduce intracellular antioxidant enzymatic activity accompanied with altered morphology and decreased viability of nasal epithelial cells. PM_2.5_ may induce oxidative stress and inflammatory responses in human nasal epithelial cells, thereby leading to nasal inflammatory diseases [6]. In the present study, we observed that the cytokines expressed after exposure to PM_2.5_ were crucial markers of the inflammation response, which included increased levels of three proinflammatory cytokines (IL-6, IL-8, and TNF-α). Therefore, infiltrating inflammatory cells and systemic inflammation may be involved in causing nasal lesions after PM_2.5_ exposure.

Our data indicated that there was decreased T-SOD activity, elevated MDA levels, and inflammatory cytokines in rat nasal mucosa exposed to PM_2.5_, which supported the viewpoint that ROS may be a consequence of the inflammatory response in nasal injury and the inflammatory response induced by PM_2.5_ exposure was related to nasal injury. Mitochondria that are associated with a source of intracellular ROS have critical roles in the regulation of cell survival and inflammation [16]. Mitochondria functions that are associated with energy production through oxidative phosphorylation and dynamic responsive sensing systems are becoming increasingly important. Mitochondrial dysfunction played an increasingly important role in disease pathogenesis and/or progression. Our work demonstrated PM_2.5_-induced morphological alterations in mitochondria, such as swelling, cristae disorder, vacuolation, and mitochondrial fission in the ultrastructural experiment. The ultra-structural damage to mitochondria subsequently had an influence on the function of the mitochondrial respiratory chain that is associated with the production of ROS and led to mitochondrial damage.

Mitochondrial damage is compensated for by fusion and eliminated by fission. The mitochondrial fusion and fission are proposed to reconcile two competing processes. Our data suggest that enhanced expression of OPA1 and Mfn1 in the nasal mucosa mitochondria can result in dilation of the mitochondria with disordered cristae, and these effects appeared under the low and moderate PM_2.5_ exposure conditions (200 and 1000 μg/m^3^). The swollen mitochondria may lead to mitochondrial membrane dysfunction and rupture. Fusion allows mitochondria to compensate for defects by sharing components, and the compensation helps maintain energy output in the face of stress as long as the stress is below a critical threshold [7]. As such, above the critical threshold could explain the decreased levels of fusion genes in high PM_2.5_ exposure conditions (3000 μg/m^3^). The significantly increased Drp1 and Fis1 expression under high PM_2.5_ exposure conditions (3000 μg/m^3^) may promote the mitochondria fission process. However, no impact of low and moderate concentrations of PM_2.5_ (200 and 1000 μg/m^3^) on fission gene expression was found in the experiment. The irreversibly damaged mitochondria are segregated by fission, which can result in abnormal alterations in mitochondrial morphology, such as fragmented and scattered mitochondria. The findings agreed with the TEM images (Figure 6). Mitochondrial dynamics may be a widespread phenomenon, but it seems these processes are different in various cell types based on cell physiology [17].

Adenosine triphosphatases (ATPases) are lipid-dependent membrane-bound enzymes that are involved in properly maintaining the transmembrane electrochemical gradients in mitochondria. Na^+^K^+^-ATPase is a sodium–potassium pump that is not only involved in the transport of Na^+^ and K^+^ ions across the mitochondrial membrane but also regulates the Ca^2+^ concentration in the mitochondria [18]. Failure of the Na*^+^*-K^+^ pumps causes the mitochondria to swell up and lyse due to uncontrolled Na^+^ influx and K^+^ efflux [19]. Mitochondrial Ca^2+^-ATPase (or the calcium pump) is responsible for the transport of the Ca^2+^ ions that maintain calcium homeostasis at the expense of ATP by hydrolysis [20]. Mitochondrial Na^+^K^+^-ATPase and Ca^2+^-ATPase activities are sensitive indicators of the structural and functional impairment of mitochondria. Significantly decreased enzyme activity of mitochondrial Na^+^K^+^-ATPase and Ca^2+^-ATP were observed in PM_2.5_ exposure groups compared to the control group. This result suggests that dysfunction of the sodium–potassium pump and calcium pump induced by PM_2.5_ can cause a homeostatic ion imbalance and mitochondrial membrane injury. The mitochondrial swelling, outer membrane rupture, or fragmentation are associated with ROS, Ca^2+^ overload, and decreased mitochondrial membrane potential [21]. Our data, which indicates that enzyme activity decreased for SOD and the levels of MDA increased, emphasizes the role of oxidative stress as a key event in mitochondrial structure and functional damage. Further studies are needed to reveal the detailed mechanisms responsible for mitochondrial dysfunction.

## 5. Conclusions

In summary, the present study demonstrates that inhalation exposure to three different concentrations of PM_2.5_ induced alterations to the main parameters oxidative stress and nasal mitochondrial dysfunction characterized by histopathological and ultrastructural damage, especially at high concentrations (3000 μg/m^3^). Our data suggests that exposure to PM_2.5_ had the potential to induce toxicity as well as oxidative damage, the inflammatory response, and mitochondrial dysfunction in the nasal mucosa of rats. This study may provide some clues to understanding the mechanisms underlying PM_2.5_-induced mammalian nasal toxicity.

## Figures and Tables

**Table 1:** Primers sequences used for real time-PCR reactions.

Symbol	Primer (5′ to 3′)
β-actin	Forward: 5′-CCTCTATGCCAACACAGT-3′ Reverse: 5′-AGCCACCAATCCACACAG-3′
OPA1	Forward: 5′-CAGCTGGCAGAAGATCTCAAG-3′ Reverse: 5′-CATGAGCAGGATTTTGACACC-3′
Mfn1	Forward: 5′-CCTTGTACATCGATTCCTGGGTTC-3′ Reverse: 5′-CCTGGGCTGCATTATCTGGTG-3′
Drp1	Forward: 5′-CGTAGTGGGAACTCAGAGCA-3′ Reverse: 5′-TGGACCAGCTGCAGAATAAG-3′
Fis1	Forward: 5′-AAATGATGCTACGCAGGCTT-3′ Reverse: 5′-CCTGGACCATGACCAAGTTT-3′
